# Peer Support Workers in Mental Health Services: A Qualitative Exploration of Emotional Burden, Moral Distress and Strategies to Reduce the Risk of Mental Health Crisis

**DOI:** 10.1007/s10597-024-01370-8

**Published:** 2024-10-17

**Authors:** Justyna Klingemann, Halina Sienkiewicz-Jarosz, Bartłomiej Molenda, Piotr Świtaj

**Affiliations:** 1 Department of Sociology of Health and Addiction Research, Institute of Psychiatry and Neurology, Sobieskiego 9, Warsaw, 02-957 Poland; 2 First Department of Neurology, Institute of Psychiatry and Neurology, Warsaw, Poland; 3 Institute of Psychiatry and Neurology, District Sanitary-Epidemiological Station, Grójec, Warsaw, Poland; 4 Maria Sklodowska-Curie Medical Academy, Warsaw, Poland

**Keywords:** Peer support work, Moral Distress, PSWs, Poland, Mental health services, Experts by experience

## Abstract

This research aimed to explore the experience of emotional burden among peer support workers (PSWs) in mental health care in Poland. It also examined the issue of moral distress in relation to this professional group and identified institutional sources of support for the well-being of PSWs in the workplace. The data presented in the article are derived from fourteen qualitative in-depth individual interviews with PSWs employed in four mental health centres with different organisational structures. The narratives of PSWs revealed several experiences that could be considered to be moral distress. The inability to assist patients was found to be associated with both individual and institutional barriers. Furthermore, our findings suggest that organisations can implement a number of specific practices to ensure the wellbeing of PSWs, which dissemination would be beneficial to teams employing PSWs.

## Introduction

The demand to base the Polish psychiatric reform on the personal recovery paradigm as a principle and *conditio sine qua non* of patient-centred psychiatric care has led to the emergence of peer support workers (PSWs) as one of the elements of the new mental health care system. The profession of PSWs is intended to act as a bridge between the patients, their family and medical staff (Davidson et al., 2012; Leamy et al., 2011; Slade et al., 2014). In Poland, a PSW may be defined as an individual who has experienced a mental health crisis themselves and subsequently undergone treatment, as well as having received appropriate training in the support of persons with mental health issues. The integration of PSWs into the therapeutic teams of Mental Health Centres (MHCs) is intended to reinforce the patients’ belief in themselves as capable of overcoming their crisis and to diminish obstacles in the patient-medical staff relationship (Chinman et al., 2014; Lloyd-Evans et al., 2014). PSWs’ personal experiences of the mental health crisis and of their own stays in psychiatric wards create conditions for ontological closeness, resulting from a sense of community of perspectives, which enhances their ability to empathise with and understand patients’ specific difficulties related to psychiatric treatment (Davidson et al., 2012). In Polish MHCs, PSWs provide support to patients in the wards by motivating them to cooperate and actively participate in treatment, accompanying them during community visits, assisting them in resolving daily problems, and facilitating access to social support. In Europe, PSWs fulfil a similar role in several other countries, including Denmark (Kirkegaard & Andersen, 2022), Finland (Kivistö et al., 2023), Germany (Otte et al., 2020), Switzerland (Burr et al., 2020; Hegedüs et al., 2016), Sweden (Wall et al., 2022) and the UK (Gillard et al., 2022). While the primary functions and responsibilities of PSWs within metal health care systems vary according to context and country, the objective of this service is consistent: to deliver recovery-oriented services, based on the personal experiences of patients, that are responsive to individual patient needs and preferences (Stefancic et al., 2019). However, the efficacy of these approaches is a matter of contention. Ongoing studies have identified a number of methodological concerns, and the results do not clearly demonstrate improvements in clinical outcomes (Chinman et al., 2014; Cooper et al., 2024; Gillard et al., 2022; Lloyd-Evans et al., 2014; White et al., 2020). Furthermore, the potential unintended consequences of the professionalisation of PSWs are also addressed (i.e. Adams, 2020; Roennfeldt & Byrne, 2021).

In addition to an analysis of the factors that facilitate and impede the implementation of this professional role in clinical practice (Ibrahim et al., 2020; Mutschler et al., 2022), the scientific literature also expresses concern about the consequences of working in this extremely psychologically demanding professional environment. Some mental health experts have expressed concern that PSWs may be unable to cope with challenging situations, which could disrupt their own recovery process and increase the likelihood of a mental health crisis (Bartosiewicz-Niziołek et al., 2021; Felton et al., 2023; Moran et al., 2013; Weikel & Fisher, 2022). This can be attributed to the high prevalence of occupational stress and burnout amongst staff engaged in mental healthcare (Bridgeman et al., 2018; Łuczak et al., 2018; O’Connor et al., 2018), as well as to the issue of compassion fatigue identified among PSWs (Steenekamp & Barker, 2024).

Nevertheless, there has been little debate surrounding another adverse phenomenon associated with the practice of psychiatric care, namely moral distress. This is a complex and multi-faceted concept, which may be defined as a set of feelings of frustration, sadness, anger or fear experienced by healthcare professionals when they encounter personal or institutional obstacles to performing what they believe is (morally) right (Deschenes et al., 2021). It has been demonstrated that moral distress can contribute to the development of depressive or anxiety disorders, sleep disorders, and professional burnout (Deschenes et al., 2021; Morley et al., 2021), or to the phenomenon known as moral injury (Čartolovni et al., 2021). In the context of the medical field, moral distress is typically discussed in relation to nurses’ work, given the frequency and intensity of contact with patients in their daily work and the limited decision-making authority nurses typically possess within the medical team (Deschenes et al., 2021; Morley et al., 2021). For these reasons, the risk of moral distress may also be high among PSWs employed in mental health care. Their position in the team hierarchy is relatively low, and they are in frequent contact with patients, often attempting to assist those who refuse treatment.

The research on strategies and interventions that effectively reduce the level of moral distress experienced by medical staff is scarce and subject to a number of methodological limitations (Deschenes et al., 2021; Morley et al., 2021). This is also due to the complexity and subjectivity of both the causes of this phenomenon and its effects. With regard to PSWs employed in mental health care, these issues have not yet been the subject of scientific analysis. In this context, it is crucial to identify the institutional sources of support used by PSWs, which could contribute to the dissemination of good practice in this area.

This research aimed to explore the experience of emotional burden among peer support workers (PSWs) in mental health care in Poland. In addition, the article explored the issue of moral distress in relation to this professional group and identified good practice in institutional sources of support for the well-being of PSWs in the workplace and to reduce the risk of mental crisis.

## Methodology

### Research Sample

The research sample consisted of 14 PSWs recruited from four MHCs located in different parts of Poland. The MHCs were selected to represent not only a range of geographical locations but also a diversity of organisational structures, including those run within psychiatric hospitals, general hospitals, research institutes with clinical base, and non-governmental organisations. A minimum of three PSWs was employed in each MHC, with three or four PSWs from each centre participating in the study. The study participants were divided equally between males and females. On average, the PSWs worked 28 h per week. The majority of respondents (86%) fell within the age range of 41–65, although it is important to note that the role of PSW is relatively new, as is the concept of MHC. Consequently, the respondents had accumulated an average of three years of work experience in this role. Of the respondents, 36% were employed on a full-time basis, 28% on a three-quarter time basis, and 36% on a half-time basis. Half of the respondents (50%) combined working in different forms of care (outpatient clinics, day ward, community treatment team, inpatient ward), while the others worked exclusively in one form of care, namely inpatient (21%) or day psychiatric ward (14%), or outpatient facilities (14%).

### Data Collection

The data for the study were collected over a two-month period (July-August) in 2022. The interviews were conducted by one of the authors of this article at each of the four MHCs. The average duration of the qualitative in-depth individual interviews with PSWs was 92 min (SD = 23.1). The interview guidelines included questions pertaining to two key areas: firstly, the burden of workload, and secondly, coping strategies. In relation to the former, the following were explored: the difficult or unforeseen situations encountered when working with patients, and how these are managed; to what extent patient narratives and the amount of information provided are considered burdensome. In terms of the latter, the following areas were addressed: how mental workloads are managed; support received in the event of challenging relationships with patients or colleagues; strategies for maintaining self-care and achieving work-life balance. All interviews were audio-recorded and transcribed (audio) following the participants’ consent.

### Data Analysis

The data analysis process involved the use of full interview transcripts (verbatim). These transcripts were coded sentence-by-sentence using a keyword approach (open coding procedure). In order to adopt a flexible and interpretive approach to the data while ensuring the reliability and systematic nature of the coding process for the extensive interview transcripts, computer-assisted qualitative data analysis (CAQDAS) was chosen. The software ATLAS.ti (version 22.2.4) was employed for this purpose. The next stage involved the organisation of the keywords associated with the selected excerpts from the analysed text into a two-level structure. This was done with the aim of assigning codes that referenced both the interview dispositions and concepts corresponding to the thematic parts of the text. The outcome of this process was the generation of the primary categories, presented in the typology discussed in the article.

### Ethical Considerations

The study was approved by the Bioethics Committee at the Institute of Psychiatry and Neurology in Warsaw (Resolution No. 16/2022). All study participants were informed that their participation was entirely voluntary. Prior to providing informed consent, participants were presented with an information sheet that outlined the objectives of the study, the methodology employed, the potential benefits and risks associated with participation, and the procedures in place to ensure the confidentiality of the data collected. Additionally, the information sheet detailed the option to withdraw from the study at any stage, without providing a reason.

### Findings

The following section presents the results of the study in relation to the perceptions and experiences of PSWs with regard to their sense of workload in MHCs and difficult or unforeseen situations related to the care of people experiencing mental health crises. Furthermore, the study identifies the repertoire of coping strategies employed by PSWs, with a particular focus on institutional sources of support. The dimensions identified in the study are illustrated with quotes labelled with information about the respondents. This includes gender (M– male/F– female), id of the respondent, the form of care in which the PSW is employed (inpatient or day ward, outpatient or community care, all/mixed), scope of employment (full-time or part-time) and years of work experience.

### Emotional Burden and Resilience

The PSWs participating in the study acknowledge that working with patients experiencing mental crises is undoubtedly emotionally demanding. They are exposed to situations where patients may have suicidal thoughts or attempts, or may become aggressive. Furthermore, working on a psychiatric ward is fraught with various unforeseen situations.

**Suicidal thoughts**

*(…) If someone talked about suicide*,* I wouldn’t want to get into it because I would worry I wouldn’t be able to cope or feel guilty afterwards. (…) (…) There was a time when a patient said he was suicidal and that if he left the support group he would harm himself. I had to get together*,* alert whoever needed to be alerted and the patient was admitted to see a doctor and a psychologist.* (M4_mixed setting_part-time_4 years’ work experience)

**Suicide**

*Maybe I’ll tell you what’s been the hardest for me*,* and I don’t think I could have coped without psychological help. I had two suicides among patients. (…) It certainly gives you a sense of what a responsible job it is and that I will never be sure when it comes to patients. I can fight for them*,* I can try*,* I can’t quite anticipate that moment.* (M11_mixed setting_part-time_3 years’ work experience)

**Aggression from patients**

*There was one situation where a patient actually went into mania and it was dangerous and I had to lock myself in the room*,* but that only happened to me once.* (F8_inpatient_full time_5 years’ work experience)

**Unforeseen situations**

*I accompanied some patients to the hospital canteen as part of the assisted outings. One patient suddenly became pale and reported that she was about to faint. I asked for help. A member of the hospital staff assisted me in leading that patient to the treatment room. I then lost track of everything that was happening. Some patients stayed in the canteen*,* some went with me. There was an open door for a while*,* so if someone just had a strong urge to escape*,* they could. This made me think*,* so we started taking patients out with two people assisting.* (M10_inpatient_full time_2 years’ work experience)

Psychiatric care is an emotionally demanding field of work, and this is reflected in the literature, which expresses concern that the recovery process of PSWs may be jeopardised as a result of the stress experienced. PSWs themselves have highlighted that they recall the beginning of their professional careers in particular as being particularly challenging, with the stress associated with a new professional role being at its highest in the first months.

(…) *The first few weeks and months were pretty chaotic*,* so I didn’t really know what I was getting myself into. I was so overwhelmed by the amount of information I had to take in about one patient that I made a mistake. Instead of going down in the lift*,* I went up to the ward above. I walked down that ward and at one point I thought I had fallen into a psychosis because it was quite different. The ward looked different*,* everything was different*,* and there were strange people there. It was such an emotional experience because it was the first time someone had told me so many things.* (M5_mixed setting_part-time_1 year’ work experience)

Concurrently, a number of respondents indicated that they had developed the capacity to work with dedication, while simultaneously prioritising their psychological well-being. This approach involved a focus on the patient, while maintaining a work-life balance.

*It’s about making sure work isn’t a burden. A good rescuer is a healthy rescuer*,* so I try to use my skills to find out what the situation is*,* what the patient has been through*,* while focusing on being empathetic and helping and supporting. I don’t get into details about what happened to the patient because that doesn’t help the work. I don’t hide the fact that I take some problems home with me. I think about it after work*,* but more in terms of how to help*,* what to do*,* and what I’ll do tomorrow.* (F8_inpatient_full time_5 years’ work experience)

*I’m not too burdened by these stories. I think I’m pretty good at striking a healthy balance*,* just like the doctors. I’m really committed to my work*,* but I try not to let it affect my personal life.* (F9_inpatient_ part-time_1 year’ work experience.

In addition, some of the PSWs who participated in the study disclosed that their mental health had worsened, and in describing the reasons for the destabilisation of their recovery, they identified a number of accumulating burdens and the considerable stress that accompanied them. For instance, the following quote illustrates the intertwined nature of stress associated with a new professional role, a demanding workload, a challenging family situation, and limited access to social support.

*One thing is for sure: the family situation is pretty tough. I have a mother who’s ill and hasn’t been able to walk for a year. I’m an only child*,* I don’t have a husband*,* a partner or children*,* so I’m facing this alone. Another thing is that in the last month*,* we’ve been swamped with support groups. On top of that*,* we’ve had a few pretty tough cases in the hospital recently. These were completely new to me*,* and I had no idea what to do. The psychologists asked me to get involved in the therapy for these people in crisis. You have to be ready to deal with whatever comes up in your work*,* but… Running a support group where there are six to twelve people*,* each with a different kind of dilemma*,* problem*,* disorder*,* psychosis*,* and you have to manage it.* (F6_ mixed setting_part-time_ 2 years’ work experience)

### Moral Distress

A number of experiences that could be characterised as moral distress were identified in the narratives of those participating in the study, which can be understood as a combination of the individual’s values and the barriers they face in taking what they believe to be the right action (Deschenes et al., 2021). For instance, PSWs are often perceived as individuals who are distinguished by a particular ontological closeness (conceptualised as a similarity of experiences) with their patients. However, despite the best endeavours of the PSWs, not all of their interactions with their patients result in the establishment of a positive and trusting relationship, which can, on occasion, lead to challenging emotional experiences.

*(…) I remember the patient. He liked to stir things up and argue with people. He’d say that I hadn’t been through anything*,* that I didn’t have any experience. He tried to provoke and dominate me so that I’d feel smaller.* (M5_mixed setting_part-time_1 year’ work experience)

*(…) I was walking down the corridor and a patient started shouting at me that I was trying to turn her into a madwoman.* (F6_mixed setting_part-time_2 years’ work experience)

*I had a patient*,* at one point he overstepped my boundaries*,* he said we were already the best of friends*,* (…) I felt suffocated? I was afraid of this relationship and I didn’t know what to do*,* it was my colleague who told me to pass him on to someone else*. (M5_mixed setting_part-time_ 1 year’ work experience)

It should be noted that instances may arise where over-involvement and an inclination to assist others may inadvertently result in conflictual situations, or alternatively the transgression of boundaries within the relationship.

*(…) I went a bit too far. I wanted to use my contact with the estate agent to recommend that person to a patient who was really worried about his house and didn’t know what to do with it and was thinking of selling it… then I saw the aggression in the patient’s sister’s eyes*,* so I backed off.* (M1_day ward_full time_5 years’ work experience)

In certain instances, identified as moral distress, a sense of being unable to assist was found to be associated with individual obstacles, including a high degree of similarity in experience or an insufficient level of competence.

**The inability to provide assistance as a consequence of the similarity of experience**

*I’ve been dealing with some pretty tough stuff recently. A psychologist asked me to take on a patient who came here with depression after her mother passed away. Another asked me to work with a woman who doesn’t want to take care of her mother. And me in all this– I have a mother with Alzheimer’s*,* so it was a bit of a challenge. One weekend*,* I sat down and cried to myself. Because I want to help them*,* but I’m trying to make sense of it all by caring for my mother*,* who I love. It was such a shock to my system. It’s difficult when it touches on my personal life*,* like my mother or father*,* or the mental crisis of losing my job*,* or the friend who left me when I started to get sick. These things come back to me when I’m working with patients. (*F6_mixed setting_part-time_2 years’ work experience)

**The inability to provide assistance due to a lack of requisite competence**

*I had a tough time with this person who was sexually non-normative. I didn’t know how to behave in a way that would maintain a culture of communication. I felt like I was thrown in at the deep end because I’m not quite competent*. (M11_mixed setting_part-time_3 years’ work experience)

In other instances identified as moral distress, the inability to provide assistance was attributed to institutional constraints, specifically the position of PSWs within the team. The low position of PSWs within hierarchical medical teams has been identified as a source of frustration due to the limited agency and high empathy towards patients.

**The inability to provide assistance due to institutional constraints**

*I noticed that one patient was in the early stages of psychosis and that she was displaying some positive symptoms. I informed the group*,* but despite this*,* the patient became agitated and disruptive. The therapist brought it to her attention*,* and I was upset because I knew she was aggressive and shouldn’t be moved. The other patient kicked her out of the class. The psychologist said I shouldn’t have spoken up*,* but I couldn’t stand it and pointed out to this patient*,* which backfired on me because she went to complain that she had the right to kick her out. I kept seeing a sick person who needed to be left alone and taken care of after group therapy. I saw that she was suffering. It was tough because the patient who kicked her out was also in a rough spot mentally. (…) Sometimes I feel helpless– I wish I could help more. There’s a lot of passivity when it comes to home life or getting help. The patient is suffering*,* there’s a problem that’s affecting them*,* and it’s causing them to become ill. I don’t know what to do because in principle they should be able to decide for themselves.* (F2_outpatient_ full time_1 year’ work experience)

### Organisational Strategies to Reduce the Risk of Mental Health Crises

The study also identified a number of good practices to reduce the risk of mental health crises in this professional group. These strategies are organisational in nature and thus worthy of dissemination for the purpose of implementation in teams employing PSWs. These include the following strategies: (a) the development of self-awareness through psychotherapy and supervision; (b) the creation of a supportive working environment, which entails ensuring the safety of staff on the ward and providing support from other PSWs and all medical staff; (c) the establishment of informal relationships through shared space and rituals; and (d) the implementation of institutional strategies, such as part-time work, leave, and sick leave.

**The development of self-awareness**.

The initial approach to mitigating the risk of a mental health crisis resulting from an excessive workload or the accumulation of negative events related to personal and professional life is to ensure that PSWs have access to services such as psychotherapy and group or individual supervision. These services aim to support both the recovery process of the PSWs and their professionalisation.

*I’m currently undergoing psychotherapy. (…) [laughs] I bring the work home with me and I talk about what I can talk about*,* with confidentiality. I tell them that I’ve had a difficult day*,* that I’ve been stressed. It’s a bit of a help.*. (F3_outpatient_ full time_4 years’ work experience)

*I think I’m pretty good at dealing with it. I’ve got a lot of resilience*,* so I don’t feel overwhelmed by patient stories. The supervisions are a great way for me to unwind. We have them quite often. They help me to relax and get the stress off my shoulders. If I have any questions or concerns*,* I can discuss them with my supervisor. (…) One of my colleagues was feeling a bit worse*,* so we talked about it at the supervision*. (M12_mixed setting_part-time_4 years’ work experience)

**The creation of a supportive working environment**.

The second group of strategies is to provide PSWs with a supportive working environment and job security. Some respondents observed that different professional environments possess distinctive characteristics, and that working in the mental health system is arguably demanding, yet at the same time, they encounter a level of understanding and support here that would be challenging to find elsewhere in the workplace.

*I’d have to deal with a lot of pressure in any job. Stress is tough for me*,* and it’s not just this profession. It’s just how I’m wired. I feel like it’s better for me in this job because there is a friendly environment and I can talk to my colleagues about what is stressing me. In other jobs I didn’t feel like I had that space to confide in my colleagues and I didn’t have the same sense of security about sharing what was stressing me.* (F3_outpatient_ full time_4 years’ work experience)

*The first one is our boss. She says and applies that as soon as something happens*,* we can come and talk. She’s like that now*,* with a colleague in the ward. The boss said that the colleague can be as sick as she wants and her work will be waiting for her. (…) When she asks us to do something*,* she always asks if we can do it*,* if it’s going to put a strain on us mentally. We then say that we’ll either do it or that we’re afraid it might affect us*,* and then there’s a discussion.* (M4_ mixed setting_part-time_4 years’ work experience)

This group of strategies also encompasses the possibility of obtaining assistance from colleagues, including both other PSWs and other staff members of MHCs.

*We chat with other PSWs about our concerns or problems*,* we share our feelings and thoughts. If someone is on sick leave*,* the person who is at work contacts them to ask about their wellbeing. This way we support each other and exchange opinions on various topics*. (F3_outpatient_full time_4 years’ work experience)

*I can chat with other PSWs about how we feel about our roles. For instance*,* when a colleague was worse*,* she came to me. We went to see the chief doctor together so that she felt she wasn’t alone. We also went to the outpatient centre to sign up. We discussed what we needed to do quickly. I’m also a PSW for her*,* and if I have a problem*,* she’ll help me*. (M10_inpatient_full time_2 years’ work experience)

*We’ve got a great team here*,* and we’re always open to discussing things. Whether you want to talk things through one-on-one or in a group setting*,* we’re here for you. (…) From the occupational therapist*,* to the psychologist*,* to the doctor– they sometimes suggest ending a process*,* setting limits*,* being more decisive*,* avoiding certain issues*,* and also taking care of yourself.* (M1_day ward_full time_5 years’ work experience)

**The establishment of informal relationships**.

Another strategy is to organise the work within inpatient or outpatient services in such a way that informal bonds between employees are fostered. This is achieved through the provision of shared work rooms, social rooms, or team-building meetings and other team rituals.

*We*,* that is to say the PSWs plus a group of psychologists*,* have a social room where we all meet to eat*,* chat*,* ask questions and get things done. We’re all in the same room together.* (F6_mixed setting_part-time_2 years’ work experience)

*We’ve got some team-building games or ways to celebrate each other’s birthdays*,* like bringing cakes*,* fruit*,* and juices*. (M1_day ward_full time_5 years’ work experience)

**The implementation of institutional strategies**.

The final group of strategies identified is the utilisation of various legal instruments related to the working environment. Some PSWs work only part-time in order to avoid an excessive workload, while others consciously utilise leave or sick leave whenever necessary.

*I get off work at 1:30 pm*,* so I can still get something done before going to bed and back to work the next day. It might help with work-life balance.* (M4_mixed setting_part-time_4 years’ work experience)

*Sometimes I take a few days off here and there. Once I took a leave of absence*,* and at the time I felt like I had to. I was afraid that something worse might happen*. (F2_outpatient_ full time_ 1 year’ work experience)

In this context, it is similarly important to ensure that staff working in a psychiatric unit feel secure in their working environment.

*I’ve never felt like I was alone. There are paramedics on hand to help in an emergency*,* as well as occupational therapists and nurses. If something happens on the ward*,* there is an alarm that can be heard on other wards*,* and then paramedics from other wards come running if it is some serious situation.* (F9_inpatient_ part-time_4 years years’ work experience)

## Discussion

An expert may possess specialised, professional training in a particular field or have accrued specific personal experience. The role of experts by experience in healthcare is expanding and is increasingly regarded as complementary to scientific knowledge (Fox, 2024; Prior, 2003). Nevertheless, experience is inherently idiosyncratic and, therefore, not a sufficient condition for comprehending the intricacies of mental disorders, their causal factors, their consequences or their treatment options. With respect to PSWs, this experience encompasses coping with acute episodes of mental illness, the ramifications of social stigma for people experiencing mental disorders, and the experience of being hospitalised for mental illness and interacting with the wider system of support available for people suffering from such disorders (Adams, 2020; Bartosiewicz-Niziołek et al., 2021). The present study addresses the question of the emotional burden of PSWs working in psychiatric healthcare and its consequences, and examines in this context moral distress in this profession. It also identifies organisational strategies for reducing the risk of mental crisis for PSWs in the workplace.

### Strengths and Limitations

The data presented in this article come from fourteen qualitative in-depth individual interviews with PSWs employed in four MHCs with different organisational structures located in different parts of Poland. Thus, the generalisation of the conclusions of the study to entities with different organisational character is substantively justified. At the same time, it seems that a particular type of MHCs was included in the study - centres constituted in the initial stages of the reorganisation of the psychiatric care system in Poland - so it can be assumed that their therapeutic teams support the concept of recovery-oriented psychiatric care in their daily work. Also importantly, these centres employed relatively large teams of PSWs (a minimum of three people), suggesting that their presence was relatively well-established within the therapeutic team and that the PSWs were able to support each other. It can be speculated that in treatment teams not working in a personal recovery paradigm and employing only one PSW, the problems presented in the study may appear with greater clarity and the identified strategies for reducing the risk of mental health crisis may prove more difficult to implement (Moran et al., 2013).

### Results Related to Previous Findings

A review of the existing literature and our own findings indicate that PSWs are subjected to considerable pressure to meet expectations. They are portrayed as highly sensitive, empathetic, and capable of overcoming barriers that other professionals may find challenging to overcome. The results of our study indicate that a significant aspect of the PSWs’ experience in their new professional role is the realisation that they are not always able to establish a positive and healthy relationship with every patient, that they are not always able to help everyone, and that they do not always have sufficient knowledge and competence to provide this help effectively. On occasion, their excessive willingness to help is met with reluctance, aggression, or simply ingratitude. They also recognise the necessity to set limits and protect themselves. Similarly, other research has demonstrated how situations within the workplace, such as difficulties in establishing a helping relationship, taking patients’ problems home, work overload and struggling to maintain a balance between work and self-care, affect PSWs on a personal level (Felton et al., 2023; Moran et al., 2013; Tate et al., 2022).

The discrepancy between expectations and reality is a source of moral distress that extends beyond the immediate context of a specific professional situation. It is shaped by the values held by the individual in question, making this phenomenon and experience distinctive (Deschenes et al., 2021). Furthermore, it is important to note that the increasing professionalisation of the profession of PSWs and the institutionalisation of the support they provide may paradoxically constitute an additional source of tension (Adams, 2020), thus becoming another cause of the moral suffering experienced.

Researchers have identified a specific risk associated with high vulnerability and low resilience to stress, which, when combined with events such as patient suicide, can precipitate a mental health crisis. Furthermore, there is evidence in the literature that PSWs may experience confusion, distress, and difficulty when confronted with novel situations (Bartosiewicz-Niziołek et al., 2021) or when hearing stories that resonate with their own experiences (Moran et al., 2013). The findings of our study corroborate these observations, indicating that working in psychiatric care is demanding for PSWs, involving a number of challenging and stressful events and the initial months in a new professional role are particularly challenging. Furthermore, the results of our study indicate that ontological proximity to patients, defined as a similarity of experiences due to gender identity, diagnosis, life experiences, and family situation, may contribute to mental crises in PSWs. However, the reasons for mental crises in this professional group require further investigation. It appears to be related to a constellation of different factors that destabilize recovery.

It is notable that the PSWs we interviewed, both those who had experienced a relapse into a mental crisis and those without, were able to implement a range of measures that served as a safety valve to ensure their psychological wellbeing and to restore their balance when faced with challenging experiences. It is likely that the feeling of confusion and psychological strain in the first months of work is linked precisely to the lack of such strategies, which also serve as a driver for their development. Research on burnout indicates that PSWs do not experience these problems more often than other members of treatment teams (Weikel & Fisher, 2022). However, it is important to note that burnout rates in the health professions are high, with younger individuals with less professional experience being particularly at risk (McCormack et al., 2018; Maslach et al., 2001; Simionato & Simpson, 2018).

## Conclusions

In the narratives of PSWs (Deschenes et al., 2021), several experiences bearing the hallmarks of moral distress were identified. The inability to assist patients was found to be associated with both individual and institutional barriers. Consequently, it is imperative that policy makers acknowledge and seek to address the complexities associated with engaging people in recovery within the recovery process (Felton et al., 2023). In addition, the findings of the study suggest that organisations can implement a number of specific practices to ensure the wellbeing of their PSWs. Dissemination of these strategies to teams employing PSWs would be beneficial, as they are not only practical but also align with common sense institutional strategies, such as building self-awareness and a supportive working environment, which include informal team relationships. It is evident that ensuring PSWs feel safe and are able to receive support from colleagues, including both other PSWs and other team members, is a crucial aspect that has been corroborated by findings from other studies (Felton et al., 2023; Jenkins et al., 2020; Steenekamp & Barker, 2024; Tate et al. 2022).

## Data Availability

The data that support the findings of this study are available from the corresponding author upon reasonable request.

## Declarations

### Author Agreement

We certify that all authors have seen and approved the final version of the manuscript being submitted. The article is our original work, it hasn’t received prior publication and isn’t under consideration for publication elsewhere.

## References

[CR1] Adams, W. E. (2020). Unintended consequences of institutionalizing peer support work in mental healthcare. *Social Science & Medicine*, *262*, 113249. 10.1016/j.socscimed.2020.113249 32768773 10.1016/j.socscimed.2020.113249

[CR2] Bartosiewicz-Niziołek, M., Kaczmarczyk-Partyka, S., Olszewski, B. H., & Ostrowska, M. (2021). Rola i funkcjonowanie asystentów zdrowienia (Ex-In) w środowiskowym modelu opieki psychiatrycznej. *Studia Psychologica: Theoria et praxis*, *21*(2), 5–18. 10.21697/sp.2021.21.2.01

[CR3] Bridgeman, P. J., Bridgeman, M. B., & Barone, J. (2018). Burnout syndrome among healthcare professionals. *American Journal of health-system Pharmacy: AJHP*, *75*(3), 147–152. 10.2146/ajhp170460 29183877 10.2146/ajhp170460

[CR4] Burr, C., Rother, K., Elhilali, L., Winter, A., Weidling, K., Kozel, B., & Gurtner, C. (2020). Peer support in Switzerland. Results from the first national survey. *International Journal of Mental Health Nursing*, *29*(2), 212–223. 10.1111/inm.12665 31618530 10.1111/inm.12665

[CR5] Čartolovni, A., Stolt, M., Scott, P. A., & Suhonen, R. (2021). Moral injury in healthcare professionals: A scoping review and discussion. *Nursing Ethics*, *28*(5), 590–602. 10.1177/0969733020966776 33427020 10.1177/0969733020966776 PMC8366182

[CR6] Chinman, M., George, P., Dougherty, R. H., Daniels, A. S., Ghose, S. S., Swift, A., & Delphin-Rittmon, M. E. (2014). Peer support services for individuals with serious mental illnesses: Assessing the evidence. *Psychiatric Services*, *65*(4), 429–441. 10.1176/appi.ps.201300244 24549400 10.1176/appi.ps.201300244

[CR7] Cooper, R. E., Saunders, K. R. K., Greenburgh, A., Shah, P., Appleton, R., Machin, K., Jeynes, T., Barnett, P., Allan, S. M., Griffiths, J., Stuart, R., Mitchell, L., Chipp, B., Jeffreys, S., Lloyd-Evans, B., Simpson, A., & Johnson, S. (2024). The effectiveness, implementation, and experiences of peer support approaches for mental health: A systematic umbrella review. *BMC Medicine*, *22*(1), 72. 10.1186/s12916-024-03260-y 38418998 10.1186/s12916-024-03260-y PMC10902990

[CR8] Davidson, L., Bellamy, C., Guy, K., & Miller, R. (2012). Peer support among persons with severe mental illnesses: A review of evidence and experience. *World Psychiatry*, *11*(2), 123–128. 10.1016/j.wpsyc.2012.05.009 22654945 10.1016/j.wpsyc.2012.05.009 PMC3363389

[CR9] Deschenes, S., Tate, K., Scott, S. D., & Kunyk, D. (2021). Recommendations for navigating the experiences of moral distress: A scoping review. *International Journal of Nursing Studies*, *122*, 104035. 10.1016/j.ijnurstu.2021.104035 34388610 10.1016/j.ijnurstu.2021.104035

[CR10] Felton, J. W., Abidogun, T. M., Senters, K., Maschino, L. D., Montgomery, B. W., Tyson, R., Furr-Holden, C. D., & Stoddard, S. A. (2023). Peer recovery coaches perceptions of their work and their implications for training, support and personal recovery. *Community Mental Health Journal*, *59*(5), 962–971. 10.1007/s10597-022-01080-z 36595145 10.1007/s10597-022-01080-z

[CR11] Fox, J. (2024). Autoethnographic reflections on mental distress and medication management: Conceptualising biomedical and recovery models of mental health. *Community Mental Health Journal*. 10.1007/s10597-024-01230-5Advance online publication. 38389027 10.1007/s10597-024-01230-5 PMC11772374

[CR12] Gillard, S., Bremner, S., Patel, A., Goldsmith, L., Marks, J., Foster, R., Morshead, R., White, S., Gibson, S. L., Healey, A., Lucock, M., Patel, S., Repper, J., Rinaldi, M., Simpson, A., Ussher, M., Worner, J., Priebe, S., & ENRICH trial study group. (2022). Peer support for discharge from inpatient mental health care versus care as usual in England (ENRICH): A parallel, two-group, individually randomised controlled trial. *The Lancet Psychiatry*, *9*(2), 125–136. 10.1016/S2215-0366(21)00398-9 35065722 10.1016/S2215-0366(21)00398-9 PMC8776565

[CR13] Hegedüs, A., Seidel, E., & Steinauer, R. (2016). Participants’ employment status and experiences in the year after the experienced involvement training. *The International Journal of Social Psychiatry*, *62*(3), 214–220. 10.1177/0020764015623969 26801072 10.1177/0020764015623969

[CR14] Ibrahim, N., Thompson, D., Nixdorf, R., Kalha, J., Mpango, R., Moran, G., Mueller-Stierlin, A., Ryan, G., Mahlke, C., Shamba, D., Puschner, B., Repper, J., & Slade, M. (2020). A systematic review of influences on implementation of peer support work for adults with mental health problems. *Social Psychiatry and Psychiatric Epidemiology*, *55*(3), 285–293. 10.1007/s00127-019-01739-1 31177310 10.1007/s00127-019-01739-1

[CR100] Jenkins, G. T., Shafer, M. S., & Janich, N. (2020). Critical issues in leadership development for peer support specialists. *Community mental health journal*, *56*(6), 1085–1094. 10.1007/s10597-020-00569-9 10.1007/s10597-020-00569-9 PMC7222857 32034639

[CR15] Kirkegaard, S., & Andersen, D. (2022). Peer workers as emotion managers: Tight and loose enactment of mutuality in mental health care. *SSM-Qualitative Research in Health*, *2*, 100200. 10.1016/j.ssmqr.2022.100200

[CR16] Kivistö, M., Martin, M., Hautala, S., & Soronen, K. (2023). Facilitators and challenges of integrating experts by experience activity in mental health services: Experiences from Finland. *Community Mental Health Journal*, *59*(3), 540–551. 10.1007/s10597-022-01039-0 36344706 10.1007/s10597-022-01039-0 PMC9981489

[CR17] Leamy, M., Bird, V., Le Boutillier, C., Williams, J., & Slade, M. (2011). Conceptual framework for personal recovery in mental health: Systematic review and narrative synthesis. *The British Journal of Psychiatry: The Journal of Mental Science*, *199*(6), 445–452. 10.1192/bjp.bp.110.083733 22130746 10.1192/bjp.bp.110.083733

[CR18] Lloyd-Evans, B., Mayo-Wilson, E., Harrison, B., Istead, H., Brown, E., Pilling, S., Johnson, S., & Kendall, T. (2014). A systematic review and meta-analysis of randomised controlled trials of peer support for people with severe mental illness. *BMC Psychiatry*, *14*, 39. 10.1186/1471-244X-14-39 24528545 10.1186/1471-244X-14-39 PMC3933205

[CR19] Łuczak, A., Baka, Ł., & Najmiec, A. (2018). Occupational stress of medical staff at psychiatric and addiction treatment wards– a review of research. *Bezpieczeństwo Pracy Nauka i Praktyka*, *2*, 6–10. 10.5604/01.3001.0010.8528

[CR20] Maslach, C., Schaufeli, W. B., & Leiter, M. P. (2001). Job burnout. *Annual Review of Psychology*, *52*, 397–422. 10.1146/annurev.psych.52.1.397 11148311 10.1146/annurev.psych.52.1.397

[CR21] McCormack, H. M., MacIntyre, T. E., O’Shea, D., Herring, M. P., & Campbell, M. J. (2018). The prevalence and cause(s) of burnout among applied psychologists: A systematic review. *Frontiers in Psychology*, *9*, 1897. 10.3389/fpsyg.2018.01897 30386275 10.3389/fpsyg.2018.01897 PMC6198075

[CR22] Moran, G. S., Russinova, Z., Gidugu, V., & Gagne, C. (2013). Challenges experienced by paid peer providers in mental health recovery: A qualitative study. *Community Mental Health Journal*, *49*(3), 281–291. 10.1007/s10597-012-9541-y 23117937 10.1007/s10597-012-9541-y

[CR23] Morley, G., Field, R., Horsburgh, C. C., & Burchill, C. (2021). Interventions to mitigate moral distress: A systematic review of the literature. *International Journal of Nursing Studies*, *121*, 103984. 10.1016/j.ijnurstu.2021.103984 34214894 10.1016/j.ijnurstu.2021.103984

[CR24] Mutschler, C., Bellamy, C., Davidson, L., Lichtenstein, S., & Kidd, S. (2022). Implementation of peer support in mental health services: A systematic review of the literature. *Psychological Services*, *19*(2), 360–374. 10.1037/ser0000531 33793284 10.1037/ser0000531

[CR25] O’Connor, K., Neff, M., D., & Pitman, S. (2018). Burnout in mental health professionals: A systematic review and meta-analysis of prevalence and determinants. *European Psychiatry*, *53*, 74–99. 10.1016/j.eurpsy.2018.06.003 29957371 10.1016/j.eurpsy.2018.06.003

[CR26] Otte, I., Werning, A., Nossek, A., Vollmann, J., Juckel, G., & Gather, J. (2020). Challenges faced by peer support workers during the integration into hospital-based mental health-care teams: Results from a qualitative interview study. *The International Journal of Social Psychiatry*, *66*(3), 263–269. 10.1177/0020764020904764 32046565 10.1177/0020764020904764

[CR27] Prior, L. (2003). Belief, knowledge and expertise: The emergence of the lay expert in medical sociology. *Sociology of Health & Illness*, *25*, 41–57. 10.1111/1467-9566.00339 14498929 10.1111/1467-9566.00339

[CR28] Roennfeldt, H., & Byrne, L. (2021). Skin in the game: The professionalization of lived experience roles in mental health. *International Journal of Mental Health Nursing*, *30*(Suppl 1), 1445–1455. 10.1111/inm.12898 34137149 10.1111/inm.12898

[CR29] Simionato, G. K., & Simpson, S. (2018). Personal risk factors associated with burnout among psychotherapists: A systematic review of the literature. *Journal of Clinical Psychology*, *74*(9), 1431–1456. 10.1002/jclp.22615 29574725 10.1002/jclp.22615

[CR30] Slade, M., Amering, M., Farkas, M., Hamilton, B., O’Hagan, M., Panther, G., Perkins, R., Shepherd, G., Tse, S., & Whitley, R. (2014). Uses and abuses of recovery: Implementing recovery-oriented practices in mental health systems. *World Psychiatry*, *13*(1), 12–20. 10.1002/wps.20084 24497237 10.1002/wps.20084 PMC3918008

[CR31] Steenekamp, B. L., & Barker, S. L. (2024). Exploring the experiences of compassion fatigue amongst peer support workers in homelessness services. *Community Mental Health Journal*, *60*(4), 772–783. 10.1007/s10597-024-01234-1 38285087 10.1007/s10597-024-01234-1 PMC11001661

[CR32] Stefancic, A., House, S., Bochicchio, L., Harney-Delehanty, B., Osterweil, S., & Cabassa, L. (2019). What we have in common: A qualitative analysis of shared experience in peer-delivered services. *Community Mental Health Journal*, *55*(6), 907–915. 10.1007/s10597-019-00391-y 30903534 10.1007/s10597-019-00391-y

[CR33] Tate, M. C., Roy, A., Pinchinat, M., Lund, E., Fox, J. B., Cottrill, S., Vaccaro, A., & Stein, L. A. R. (2022). Impact of being a peer recovery specialist on work and personal life: Implications for training and supervision. *Community Mental Health Journal*, *58*(1), 193–204. 10.1007/s10597-021-00811-y 33677802 10.1007/s10597-021-00811-y

[CR34] Wall, A., Lovheden, T., Landgren, K., & Stjernswärd, S. (2022). Experiences and challenges in the role as peer support workers in a Swedish mental health context - an interview study. *Issues in Mental Health Nursing*, *43*(4), 344–355. 10.1080/01612840.2021.1978596 34569894 10.1080/01612840.2021.1978596

[CR36] Weikel, K., & Fisher, T. (2022). Burnout and turnover intention among peer providers and other providers of mental health services in a rural two-county area. *Journal of Psychosocial Rehabilitation and Mental Health*, *9*(1), 33–43. 10.1007/s40737-021-00232-w 34458073 10.1007/s40737-021-00232-w PMC8383238

[CR37] White, S., Foster, R., Marks, J., Morshead, R., Goldsmith, L., Barlow, S., Sin, J., & Gillard, S. (2020). The effectiveness of one-to-one peer support in mental health services: A systematic review and meta-analysis. *Bmc Psychiatry*, *20*, 534. 10.1186/s12888-020-02923-3 33176729 10.1186/s12888-020-02923-3 PMC7657356

